# Rectus Femoris Autograft Harvesting: Tips and Tricks

**DOI:** 10.1002/atn2.70279

**Published:** 2026-09-23

**Authors:** Riccardo D’Ambrosi, Andrea Mei, Rodolfo Muscarella, Alessandro Di Pace, Eno Picori, Luca Maria Sconfienza, Nicola Ursino

**Affiliations:** ^1^ IRCCS Ospedale Galeazzi ‐ Sant’Ambrogio Milan Italy; ^2^ Link Campus University Rome Italy; ^3^ Residency Program in Orthopaedic and Traumatology University of Milan Milan Italy; ^4^ Dipartimento di Scienze Biomediche per la Salute Università degli Studi di Milano Milan Italy

## Abstract

The rectus femoris tendon has recently emerged as a potential graft option for anterior cruciate ligament reconstruction because of its favorable biomechanical properties, substantial graft length, and minimally invasive harvest characteristics. As the most superficial layer of the quadriceps tendon complex, the rectus femoris tendon can be selectively harvested while preserving the deeper quadriceps layers and extensor mechanism continuity. This technical note describes a reproducible minimally invasive technique for rectus femoris tendon harvest, emphasizing surgical anatomy, dissection planes, technical pearls, and pitfalls to optimize graft quality and minimize complications.

## Graphical abstract

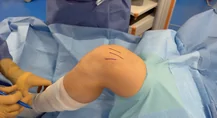

**VIDEO 1:** Left knee. The rectus femoris tendon, corresponding to the superficial layer of the quadriceps tendon, is harvested through a limited longitudinal incision located 2 to 3 cm proximal to the superior pole of the patella, between the center of the patella and its lateral border. After identification of the plane between the rectus femoris and vastus intermedius, a tendon strip is created and carefully elevated while preserving the deeper quadriceps layers. The graft is detached distally, mobilized proximally, and harvested using a closed tendon stripper. Key technical pearls include identification of the correct harvest plane approximately 3 cm proximal to the superior pole of the patella, maintenance of a strictly superficial dissection plane, and preservation of the intermediate and deep quadriceps layers to avoid capsular violation and subsequent arthroscopic fluid extravasation. The inset displayed in the lower right corner illustrates the dissection on an anatomical model of a right knee, providing a detailed visualization of the relevant anatomical planes and the harvesting technique. Video content can be viewed at https://doi.org/10.1002/atn2.70279.

Anterior cruciate ligament (ACL) reconstruction continues to evolve in the pursuit of grafts that provide optimal biomechanical properties while minimizing donor‐site morbidity and preserving postoperative function. Despite the widespread use of hamstring tendon, bone–patellar tendon–bone, and quadriceps tendon autografts, no single graft has showed clear superiority across all clinical scenarios.^1^, ^2^

In recent years, increasing attention has been directed toward the rectus femoris (RF) tendon as an alternative graft source for ligament reconstruction. As the superficial layer of the quadriceps tendon complex, the RF tendon can be harvested selectively while preserving the deeper quadriceps layers and extensor mechanism integrity. This approach allows procurement of a long soft‐tissue graft with predictable dimensions, limited violation of the quadriceps mechanism, and potentially reduced donor‐site morbidity.^3^, ^4^, ^5^, ^6^

Biomechanical evidence supporting the RF tendon has recently emerged. Mestriner et al.^5^ showed that a double‐stranded RF graft used for ACL reconstruction exhibited ultimate tensile strength and mechanical properties comparable to those of the patellar tendon graft, whereas the single‐stranded RF configuration showed biomechanical behavior similar to iliotibial band grafts used for anterolateral procedures. These findings support the feasibility of using the RF tendon for combined ligament reconstruction procedures using a single harvest site.^7^ Furthermore, anatomic studies have showed the existence of a reproducible and well‐defined superficial layer that allows safe harvest while preserving the intermediate and deep layers of the quadriceps tendon.^5^, ^6^, ^7^

From a surgical standpoint, several technical advantages have been described. The RF tendon can provide graft lengths ranging from approximately 28 to 35 cm, allowing multiple configurations for isolated ACL reconstruction, combined ACL and anterolateral ligament reconstruction, anteromedial oblique ligament augmentation, and even revision or multiligament procedures.^7^, ^8^, ^9^, ^10^ In addition, the superficial harvest may decrease the risk of arthrotomy and fluid extravasation while maintaining quadriceps continuity.^7^, ^11^

Early clinical reports have also shown encouraging results. Previous studies reported satisfactory short‐term outcomes, low harvest‐related morbidity, and near‐complete recovery of quadriceps strength within the first postoperative months after RF tendon harvest.^3^, ^4^, ^6^ Comparative investigations have further suggested functional outcomes similar to conventional autografts while preserving extensor mechanism performance.^3^, ^4^, ^6^

Therefore, the purpose of this technical note is to describe our surgical technique for minimally invasive RF tendon harvest for ACL reconstruction and highlight the technical pearls and pitfalls associated with this emerging graft option.

## SURGICAL TECHNIQUE

After administration of spinal anesthesia, a clinical examination is performed to confirm knee instability and assess ligamentous laxity (Table 1). A pneumatic tourniquet is routinely used during the procedure. The patient is positioned supine, with the knee flexed at 90° over a double bolster (Table 2). The relevant anatomical landmarks are identified and marked before skin incision.

**TABLE 1:** Instrumentation Required for Rectus Femoris Harvest

Instrumentation
Two small Farabeuf retractors
Metzenbaum dissecting scissors
No. 20 scalpel blade for skin incision
No. 15 scalpel blade for soft‐tissue dissection
O’Shaughnessy curved dissecting forceps
Kocher clamp
Sterile gauze
No. 2 Vicryl suture
Closed tendon stripper (minimum diameter: 7 mm)

**TABLE 2:** Surgical Steps

Step	Surgical Step	Key Point
1	Patient positioning	Supine position; knee flexed to 90° over a double bolster
2	Landmark identification	Mark the central patellar point, lateral patellar border, and reference line between them
3	Skin incision	Position the leg laterally at the edge of the operating table to improve exposure. Perform a 3‐cm longitudinal incision 3 to 4 cm proximal to the superior pole of the patella
4	Superficial dissection	Carefully dissect subcutaneous tissue until the quadriceps tendon plane is reached
5	Tendon identification	Identify the rectus femoris as the most superficial layer of the quadriceps tendon complex
6	Dissection plane	Use the fatty areolar layer between the rectus femoris and deeper quadriceps layers as the key landmark
7	Tendon isolation	Make 2 short vertical incisions along the medial and lateral margins of the rectus femoris tendon
8	Deep release	Pass an O’Shaughnessy curved forceps underneath the tendon to separate it from the deeper layers
9	Distal detachment	Extend the distal cuts and detach the tendon distally with a transverse horizontal cut
10	Graft control	Grasp the distal tendon with a Kocher clamp and place a No. 2 Vicryl whipstitch
11	Initial mobilization	Use blunt scissors to release the tendon distally to proximally for approximately 8 cm
12	Tendon stripping	Harvest the graft with a closed ≥7‐mm tendon stripper after the rectus femoris axis
13	Knee position during stripping	Maintain the knee at 10° to 20° of flexion to facilitate stripper advancement
14	Final inspection	Assess graft length and quality; inspect the donor site for hemostasis and capsular integrity

**FIGURE 1:**
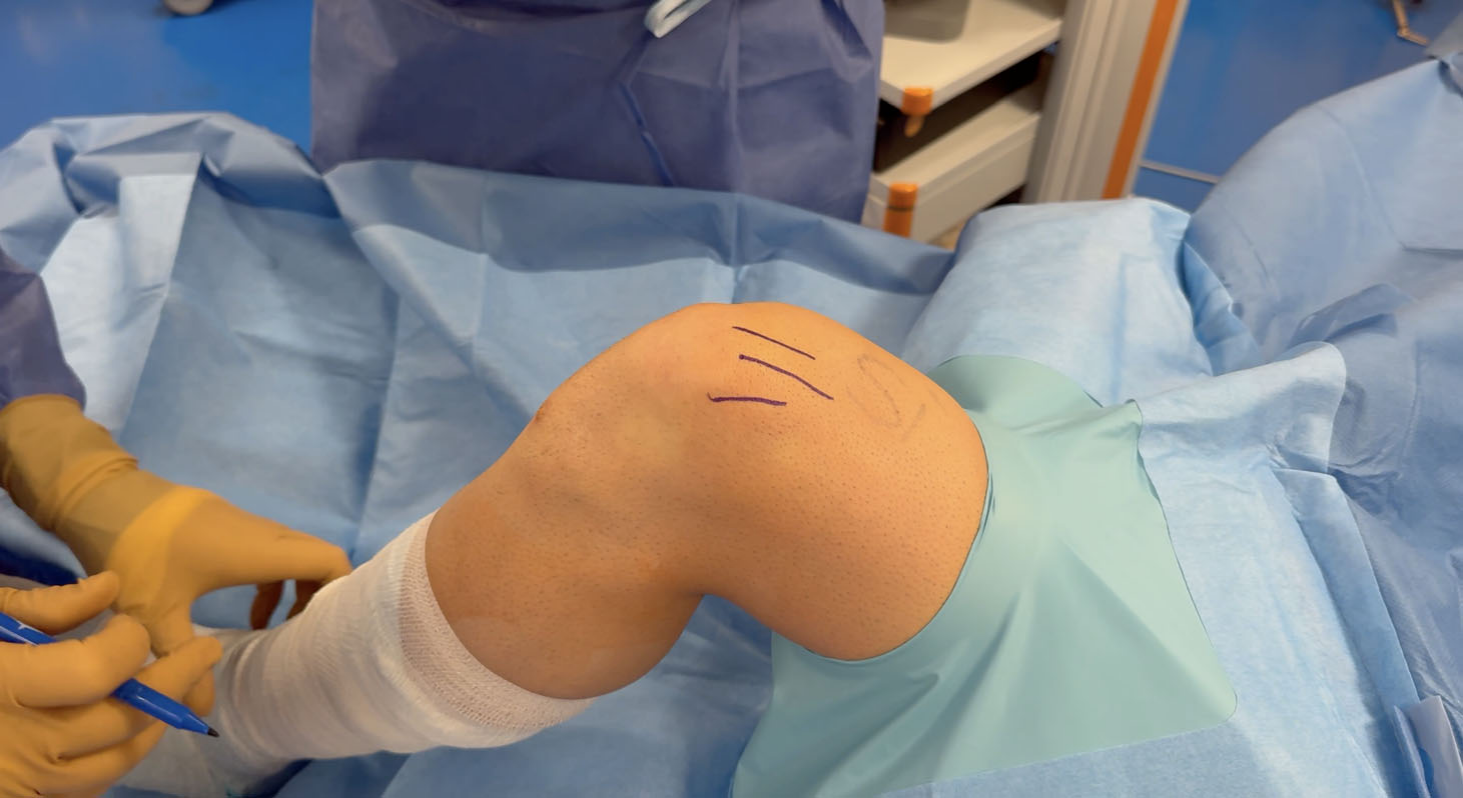
Left knee positioned at 90° of flexion. The 3 anatomical landmarks are identified and marked: the central point of the patella, the lateral patellar border, and the reference line drawn between these 2 points.

The midpoint of the patella and the lateral patellar border are marked, and a reference line is drawn between these 2 landmarks. This reference line guides graft harvesting, which is centered between the patellar midpoint and the lateral patellar border rather than directly on the quadriceps tendon midline (Figure 1).

**FIGURE 2:**
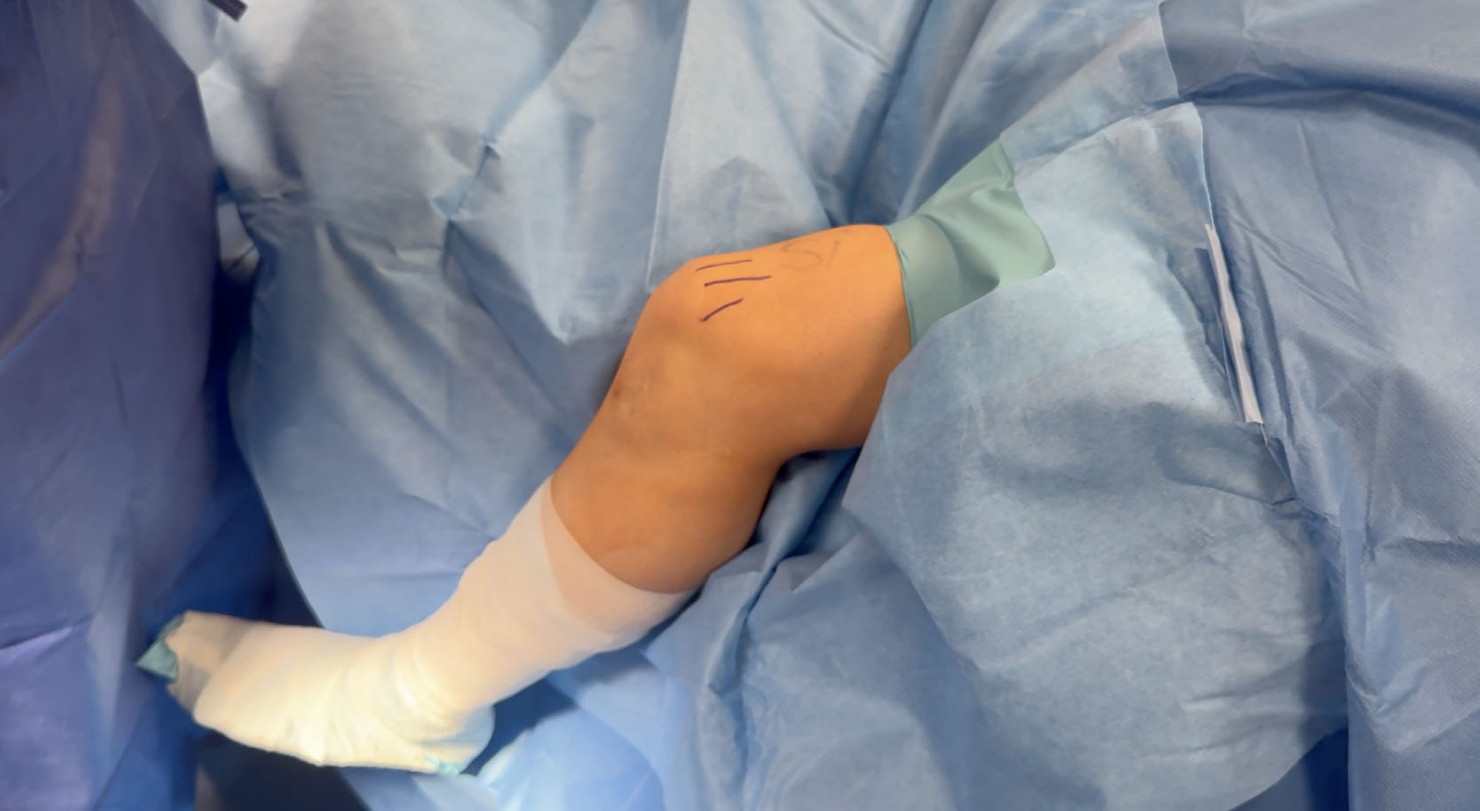
Left knee. After completion of the skin markings, the leg is positioned laterally at the edge of the operating table to improve exposure and facilitate instrumentation.

**FIGURE 3:**
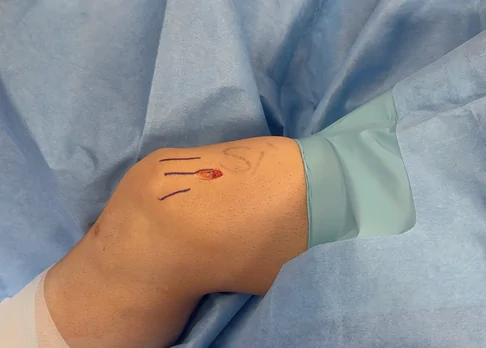
Left knee. A small longitudinal incision, approximately 3 cm in length, is performed 3 to 4 cm proximal to the superior pole of the patella after the longitudinal axis of the rectus femoris tendon and the previously drawn landmark.

**FIGURE 4:**
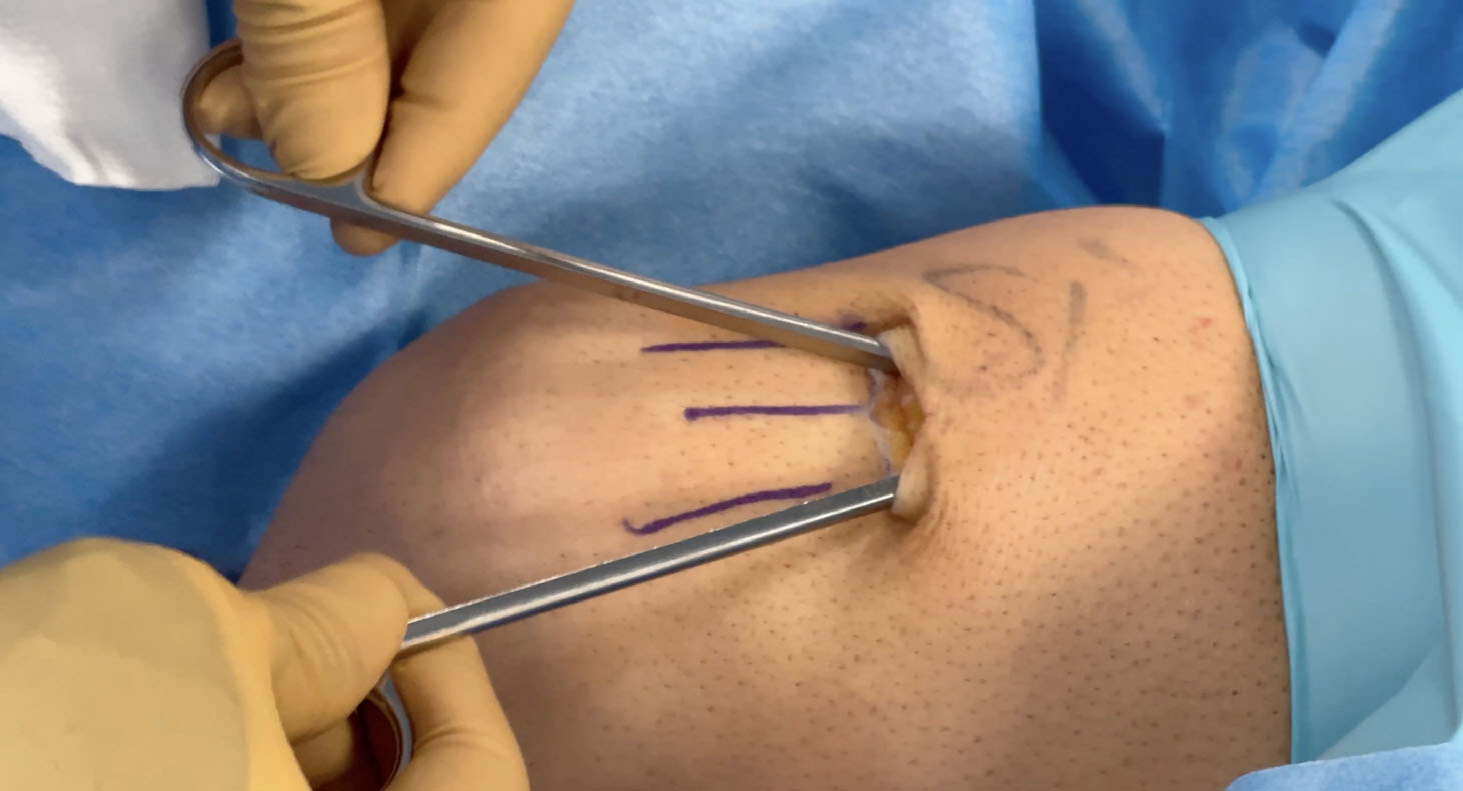
Left knee. The superficial layers and subcutaneous tissue are progressively dissected using scissors until the tendinous layer is reached.

After completion of the skin markings, the operative leg is positioned at the edge of the operating table to improve exposure and facilitate instrumentation (Figure 2). A small longitudinal incision, approximately 3 cm in length, is made 3 to 4 cm proximal to the superior pole of the patella, in line with the longitudinal axis of the RF tendon (Figure 3). The approach is kept as limited as possible. Blunt dissection of the subcutaneous tissue is performed using scissors (Surtex Instruments, New Malden, Surrey, United Kingdom) and sterile gauze until the quadriceps tendon is clearly exposed (Figure 4).

**FIGURE 5:**
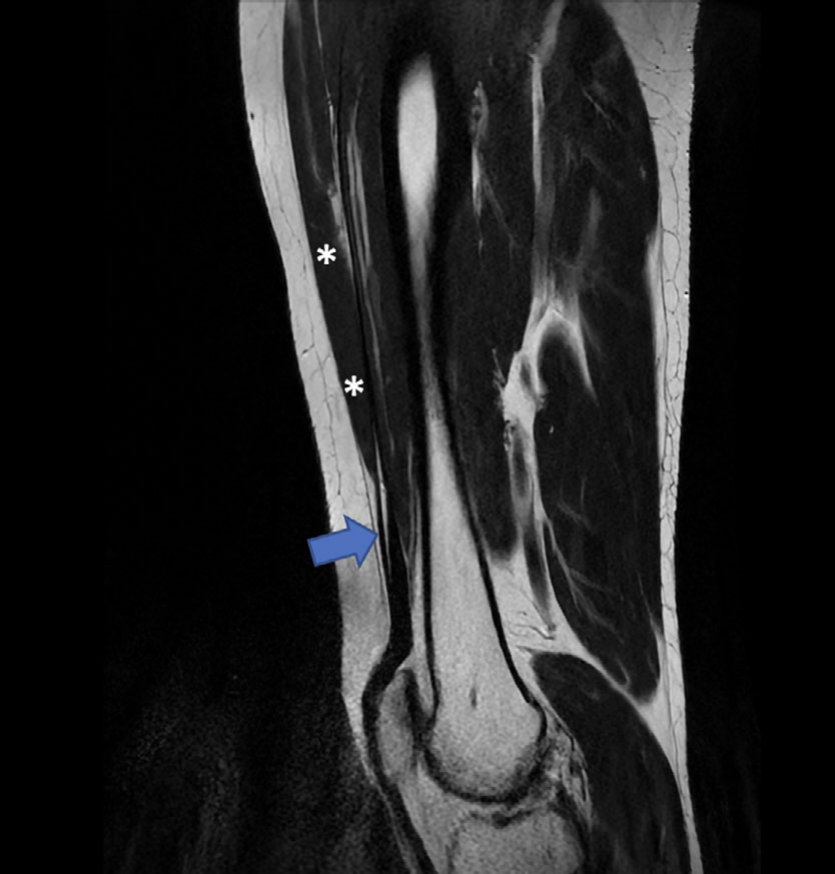
Right knee. Sagittal T1‐weighted MRI scan of the distal thigh showing the anatomical course of the rectus femoris tendon. The blue arrow indicates the distal portion of the tendon, whereas the white asterisks highlight its superficial trajectory over the deeper quadriceps layers. The image emphasizes the superficial location of the rectus femoris tendon within the quadriceps tendon complex. (MRI, magnetic resonance imaging.)

**FIGURE 6:**
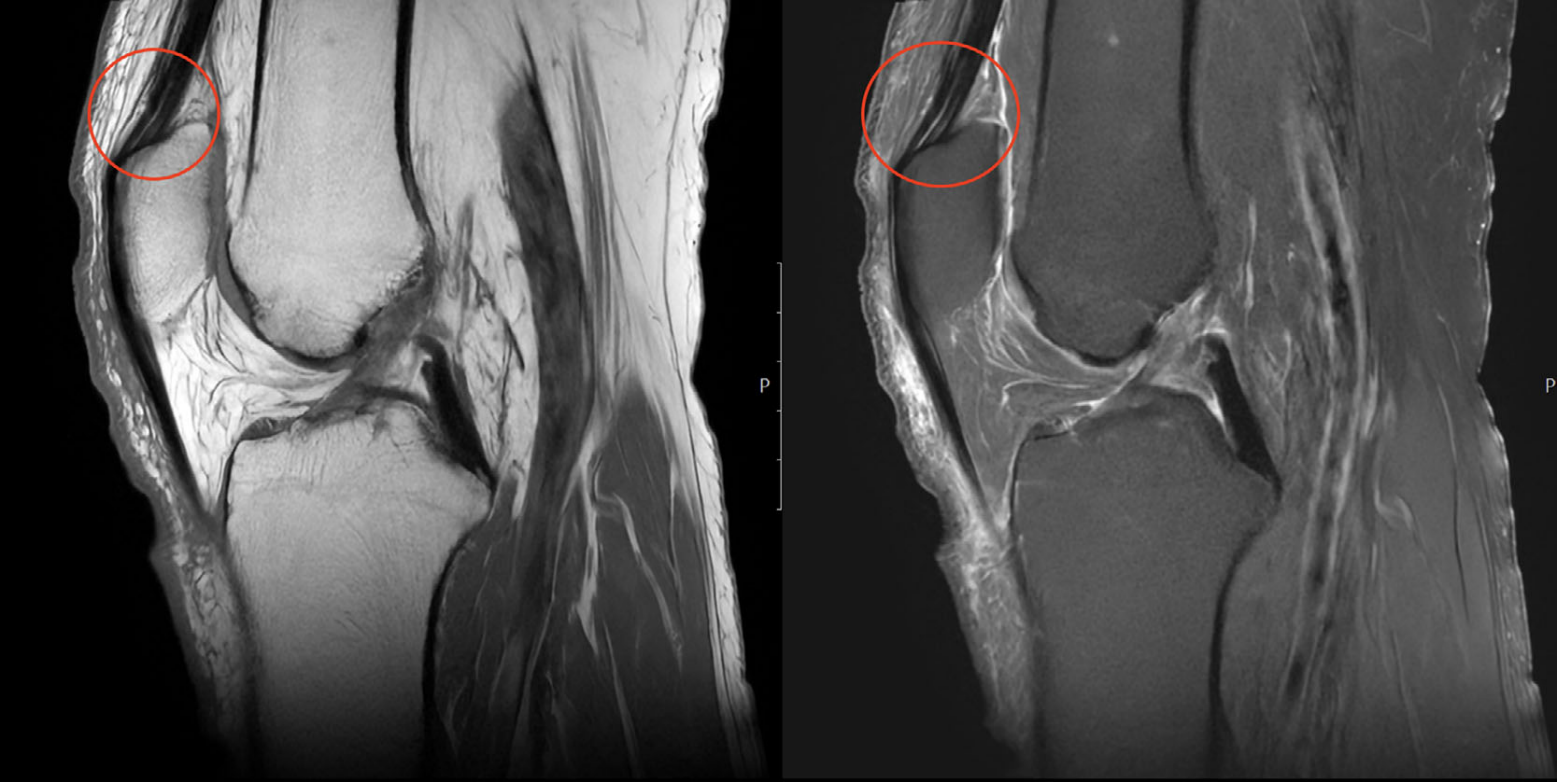
Right knee. Sagittal MRI scans of the distal quadriceps tendon complex. The left image shows a T1‐weighted sequence, whereas the right image shows a fluid‐sensitive fat‐suppressed sequence. In both images, the red circles highlight the thin fatty areolar layer interposed between the rectus femoris tendon and the deeper quadriceps tendon layers. This adipose tissue plane represents a key anatomical landmark for identification of the correct dissection plane during graft harvesting. (MRI, magnetic resonance imaging.)

**FIGURE 7:**
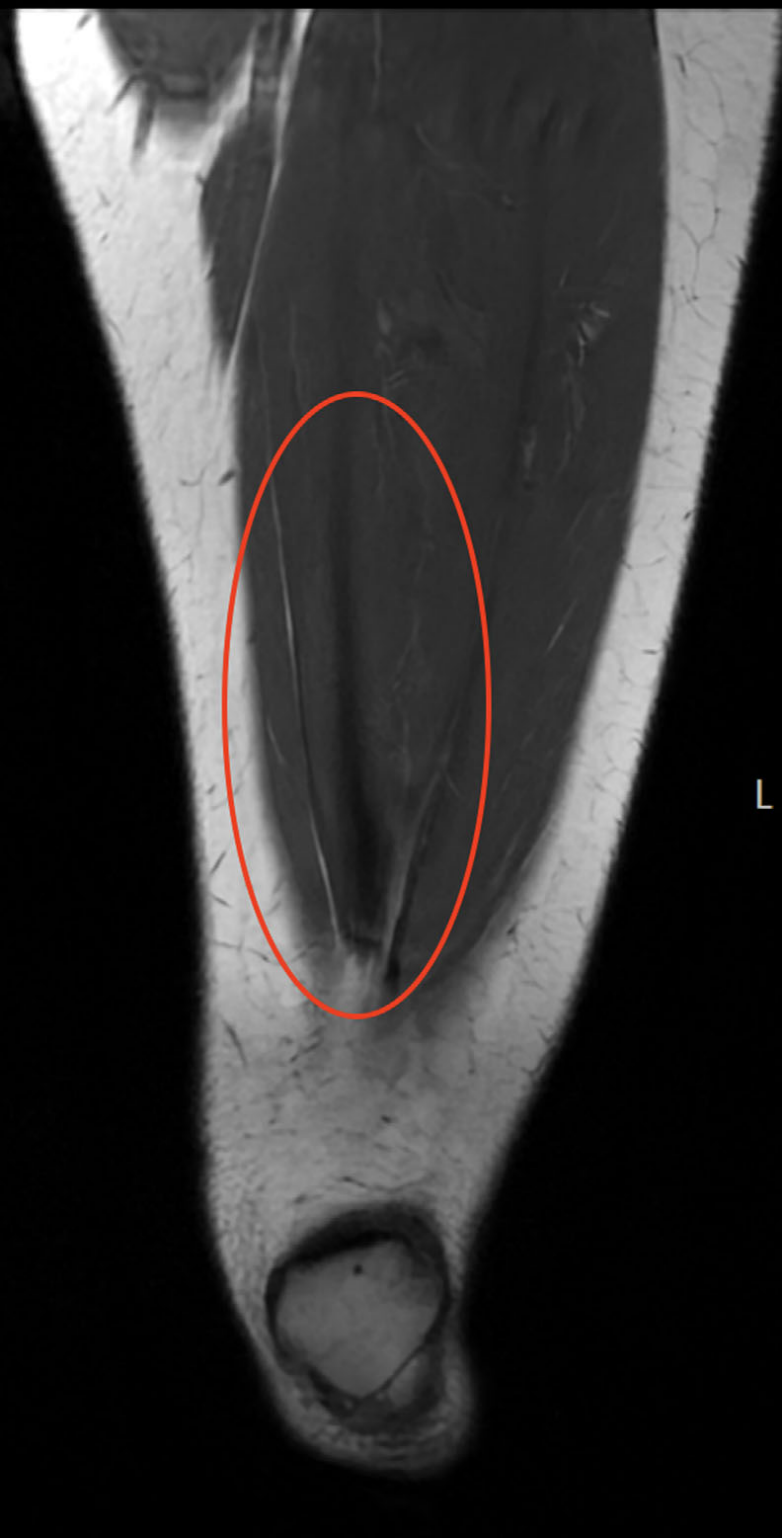
Left knee. Coronal T1‐weighted MRI scan of the distal thigh showing the anatomical course of the rectus femoris tendon. Within the red circle, the rectus femoris tendon can be identified between the 2 muscle bellies, confirming its superficial and central position within the quadriceps tendon complex. (MRI, magnetic resonance imaging.)

Once the quadriceps tendon is exposed, 2 Farabeuf retractors (Gima S.p.A., Gessate, Italy) are used to improve visualization. The RF tendon is identified as the most superficial layer of the quadriceps tendon complex (Figure 5). A thin fatty areolar plane separating the RF tendon from the deeper quadriceps layers serves as a reliable anatomical landmark for identification of the correct harvest plane (Figures 6 and 7).

The key step of the technique is identification and maintenance of the correct dissection plane between the RF tendon and the deeper quadriceps layers, particularly the deep quadriceps tendon component and the vastus intermedius. Preservation of this plane allows safe tendon isolation while avoiding violation of the suprapatellar pouch.

**FIGURE 8:**
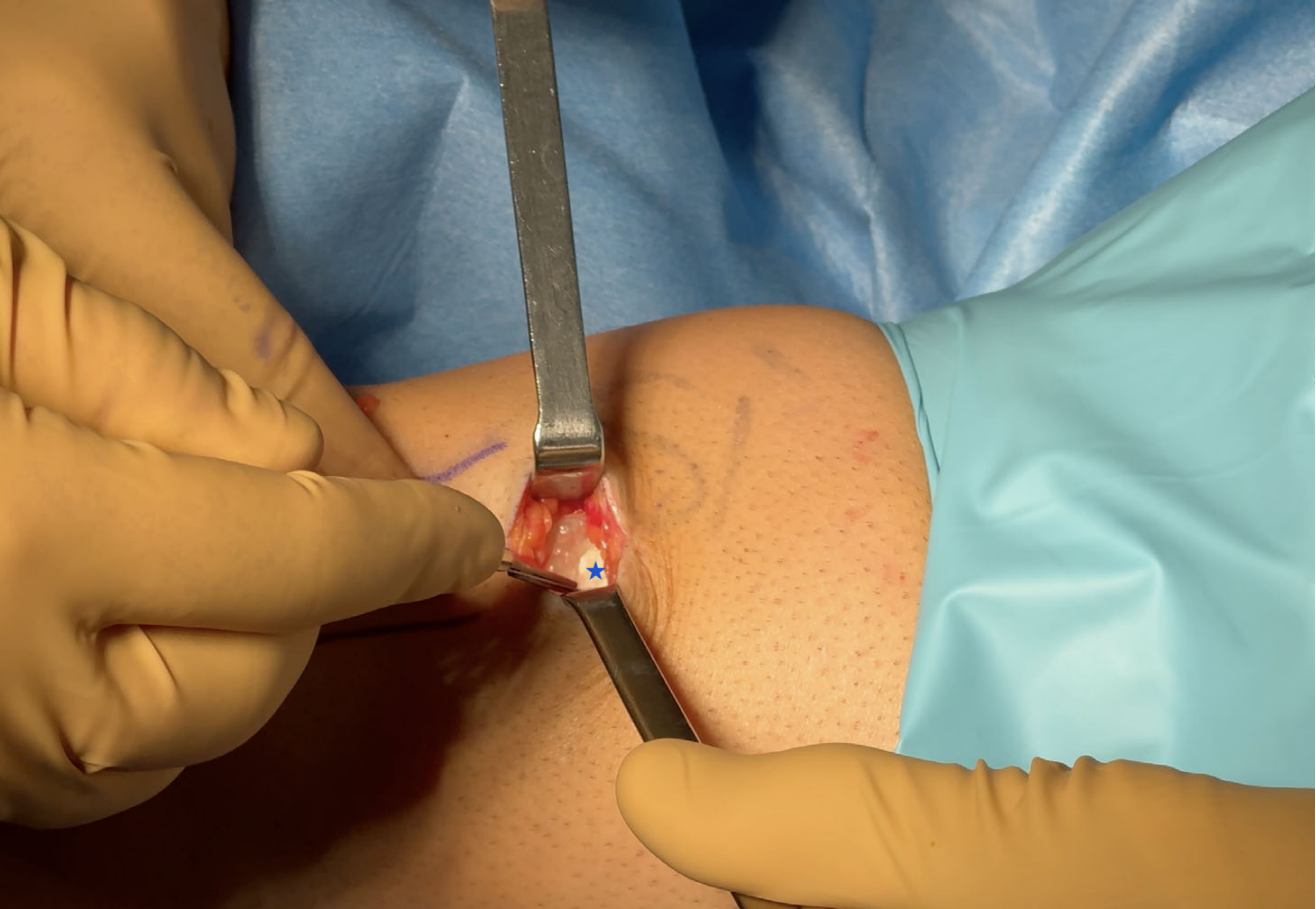
Left knee. After identification of the tendon, 2 short vertical incisions are made along the medial and lateral margins of the rectus femoris tendon. These incisions are approximately 1 cm in length and only a few millimeters deep. The rectus femoris tendon is identified by the blue asterisk in the image.

Two short vertical incisions are then made along the medial and lateral margins of the RF tendon. These incisions are approximately 1 cm in length and only a few millimeters in depth (Figure 8). The thin fatty areolar layer remains a key landmark for confirming the correct dissection plane.

**FIGURE 9:**
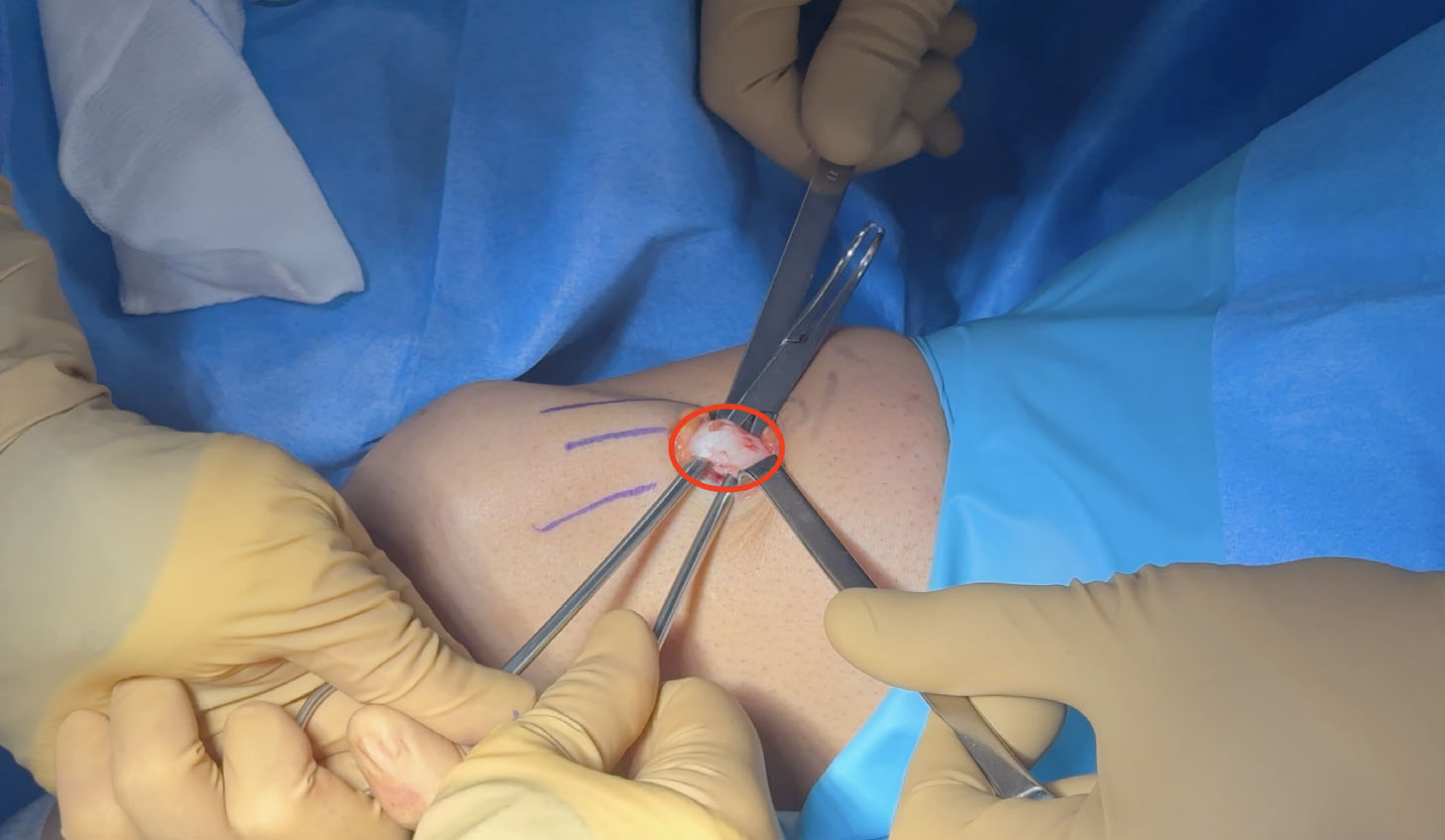
Left knee. Once the 2 vertical incisions have been completed, an O’Shaughnessy curved dissecting forceps is passed underneath the tendon to complete its isolation from the underlying layers. The red circle highlights the isolated rectus femoris tendon.

**FIGURE 10:**
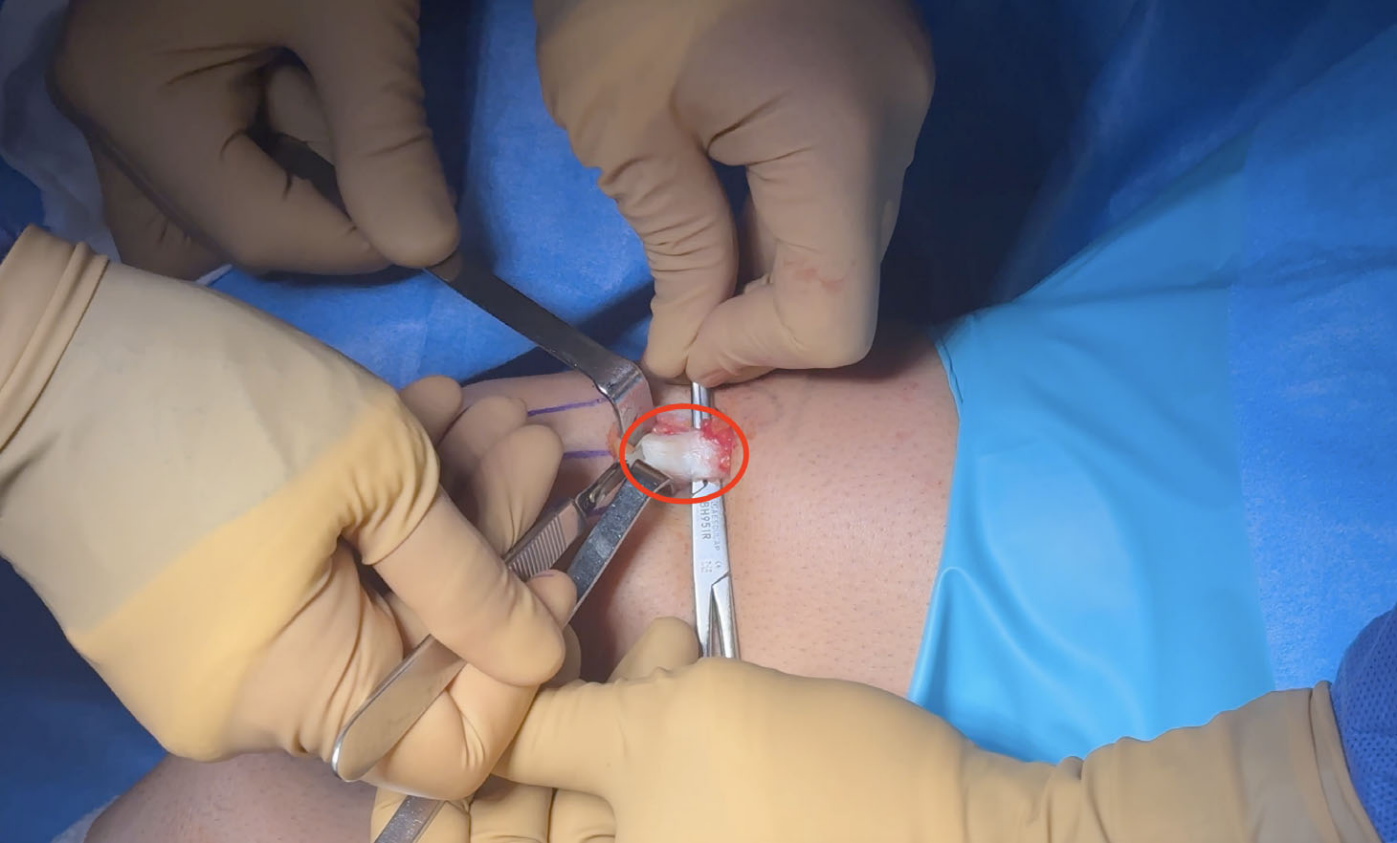
Left knee. The distal cuts are extended toward the insertional area, and the tendon is finally detached distally using a transverse horizontal cut. The red circle highlights the detached distal end of the rectus femoris tendon.

Once the 2 vertical incisions have been completed, an O’Shaughnessy curved dissecting forceps (Surtex Instruments, New Malden, Surrey, United Kingdom) is passed beneath the tendon to complete its separation from the underlying layers (Figure 9). The medial and lateral incisions are then extended distally toward the insertion site, and the tendon is detached distally with a transverse cut (Figure 10).

**FIGURE 11:**
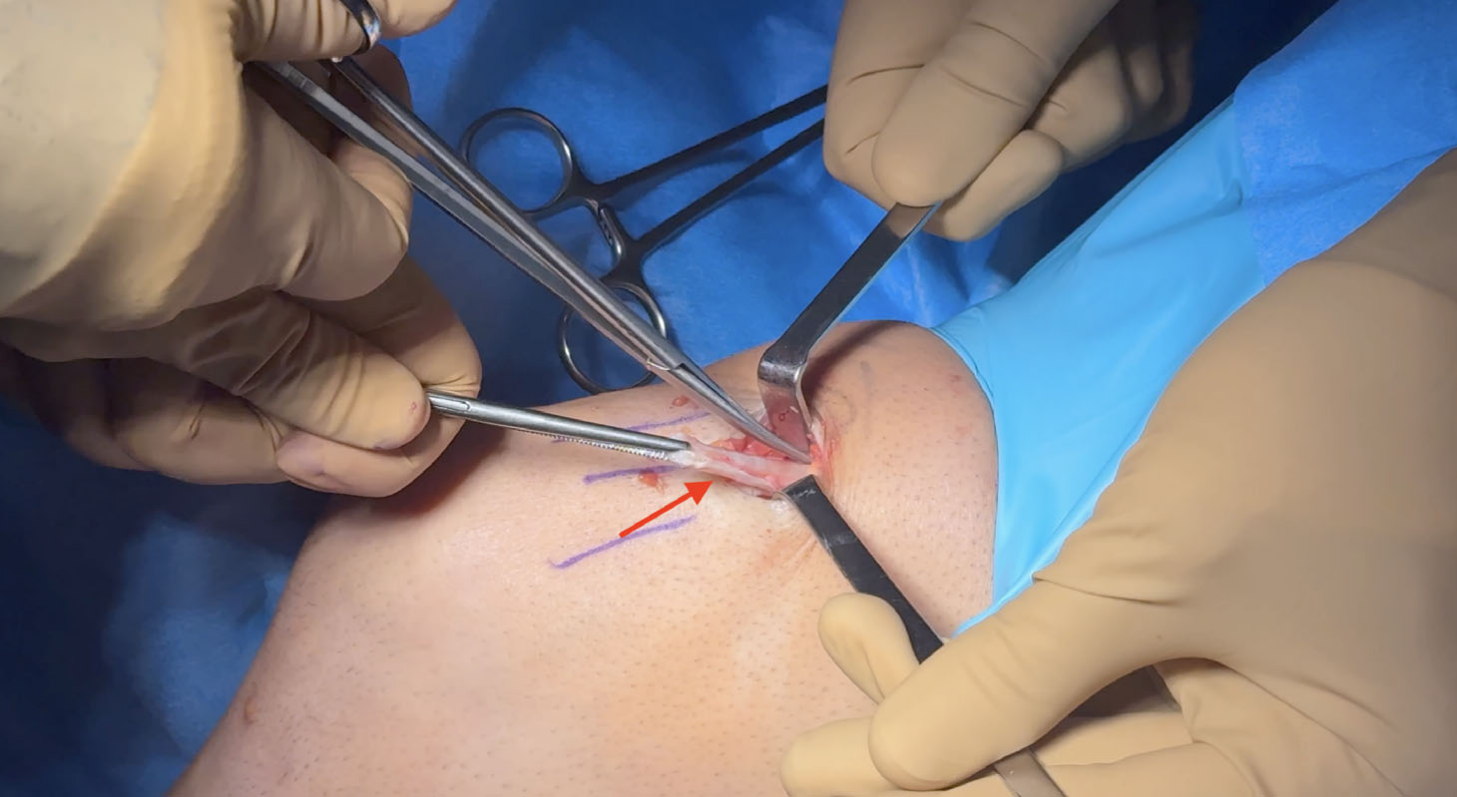
Left knee. The distal end of the rectus femoris tendon is grasped with a Kocher clamp. The tendon is indicated by the red arrow.

The distal end of the tendon is grasped with a Kocher clamp (Surtex Instruments, New Malden, Surrey, United Kingdom). With the tendon secured, blunt scissors are used to continue the dissection in a distal‐to‐proximal fashion for approximately 8 cm. This step is particularly important because the distal portion of the tendon is often more adherent and difficult to separate from the underlying tissues (Figure 11).

**FIGURE 12:**
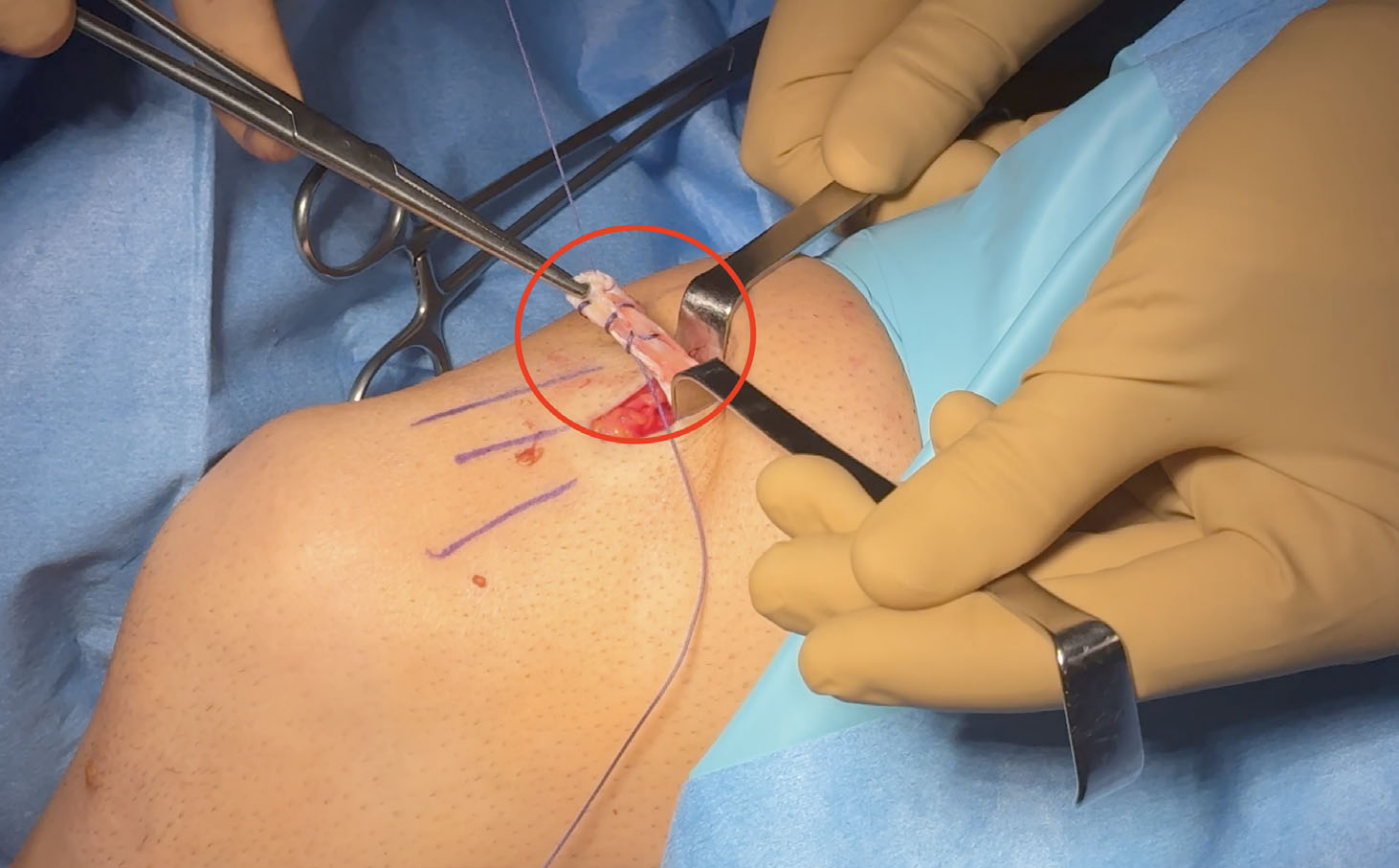
Left knee. The distal portion of the tendon is prepared using a nonabsorbable suture to allow controlled traction during the subsequent steps. The rectus femoris tendon is visible within the red circle.

A whipstitch is then placed in the distal portion of the graft using a No. 2 Vicryl (Ethicon, Raritan, NJ) suture to allow controlled traction during the subsequent steps (Figure 12). The graft is subsequently harvested using a closed tendon stripper (Arthrex, Naples, FL) with a diameter of at least 7 mm. Care must be taken to maintain the stripper along the longitudinal axis of the RF tendon.

This represents a critical step of the procedure, as deviation from the longitudinal tendon axis may compromise graft quality, reduce graft length, or result in an irregular harvest. During stripping, the knee is maintained at approximately 10° to 20° of flexion to facilitate advancement of the stripper along the dissection plane.

**FIGURE 13:**
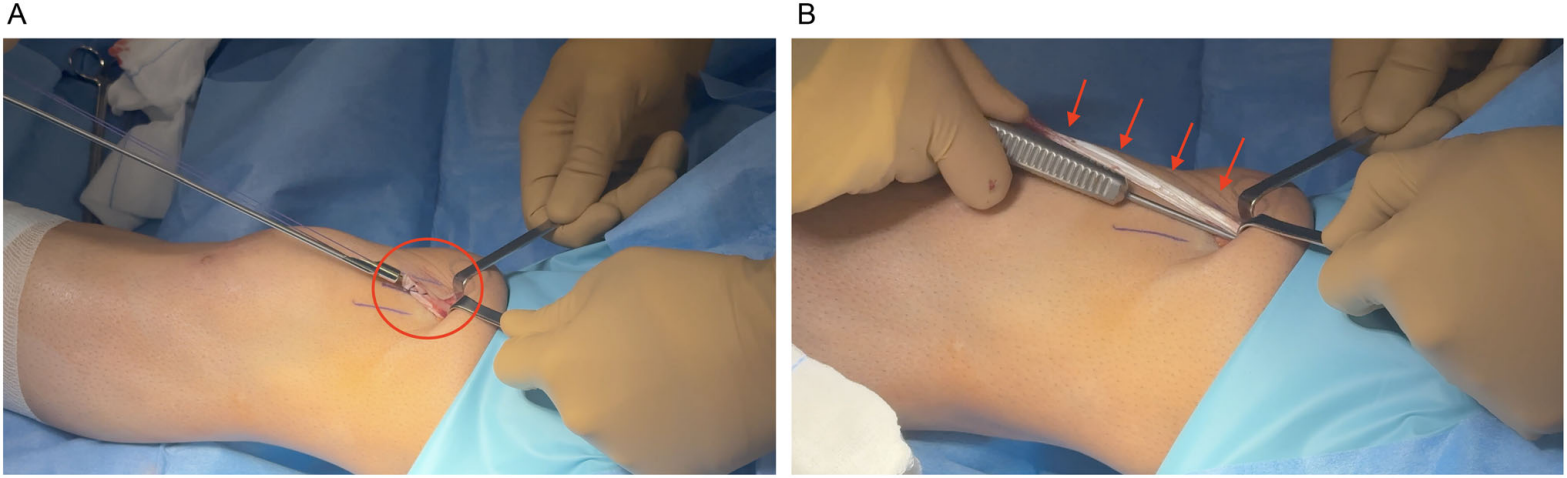
(A,B) Left knee. The graft is subsequently harvested using a closed tendon stripper. A closed tendon stripper with a diameter of at least 7 mm is strongly recommended. The stripper must follow the longitudinal axis of the rectus femoris tendon. During stripping, the knee is maintained at approximately 10° to 20° of flexion to facilitate advancement of the stripper along the dissection plane. The rectus femoris tendon is highlighted within the red circle. As shown in (B), once the proximal portion of the harvest is reached, nearly the entire tendon stripper is advanced within the thigh, confirming proximal progression along the rectus femoris tendon axis. The rectus femoris tendon is indicated by the red arrows.

With constant and controlled traction applied to the graft, harvesting is performed in a proximal direction. The stripper is advanced along the tendon axis to obtain a graft of adequate length and uniform morphology (Figure 13A,B).

Harvesting should be performed gently, avoiding excessive force and maintaining the correct dissection plane throughout the procedure. Accurate identification and preservation of this plane are essential to obtain a high‐quality graft (Video 1).

Once harvesting is complete, the tendon is extracted and assessed for length, thickness, and macroscopic integrity. The donor site is then carefully inspected to confirm hemostasis, preservation of the deeper quadriceps layers, and the absence of suprapatellar pouch violation (Table 3).

**TABLE 3:** Pearls and Pitfalls

Pearls	Pitfalls
Carefully identify the rectus femoris tendon as the most superficial layer of the quadriceps tendon complex	Incorrect identification of the tendon may result in harvesting deeper quadriceps layers
Use the thin fatty areolar layer as the key landmark to maintain the correct dissection plane	Loss of the fatty plane may lead to capsular violation or injury to the vastus intermedius
Keep the surgical approach as limited as possible to reduce soft‐tissue disruption	Excessive dissection may increase morbidity and impair visualization of the correct plane
Perform the medial and lateral tendon incisions only a few millimeters deep	Deep cuts may damage the deeper quadriceps tendon layers
Use blunt dissection with scissors and gauze during tendon mobilization	Aggressive sharp dissection may cause tendon damage or irregular graft morphology
Adequately release the distal fibrotic portion of the tendon before stripper advancement	Insufficient distal release may increase resistance during stripping and predispose to premature graft amputation
Maintain the tendon stripper aligned with the longitudinal axis of the rectus femoris tendon	Incorrect stripper trajectory may compromise graft length and quality
Maintain the knee at approximately 10° to 20° of flexion during graft harvesting	Excessive knee flexion may hinder smooth advancement of the tendon stripper
Apply constant and progressive traction during proximal stripping	Excessive force may cause graft tearing or disruption of the dissection plane
Carefully inspect the donor site at the end of the procedure	Failure to recognize capsular violation or inadequate hemostasis may increase postoperative complications

## DISCUSSION

The most important finding of this technical note is that the RF tendon can be harvested through a reproducible minimally invasive approach while preserving the integrity of the deeper quadriceps layers and providing a long, versatile soft‐tissue graft suitable for ACL reconstruction. The described technique allows reproducible identification of the superficial RF layer while maintaining the vastus intermedius and the remaining quadriceps tendon intact, thereby avoiding a full‐thickness quadriceps defect (Table 4).^7^, ^11^

**TABLE 4:** Advantages and Disadvantages of Rectus Femoris Tendon Harvest for ACL Reconstruction

Advantages	Disadvantages
Minimally invasive harvest through a small proximal incision	Learning curve for identification of the correct surgical plane between the rectus femoris and vastus intermedius
Preserves the intermediate and deep layers of the quadriceps tendon and the suprapatellar capsule	Limited long‐term clinical evidence compared with hamstring, patellar tendon, and quadriceps tendon autografts
Provides a long graft that can be prepared in different configurations (double‐, triple‐, or quadruple‐strand)	Potential transient quadriceps weakness during the early postoperative rehabilitation period
Adequate graft diameter, length, and biomechanical properties for ACL reconstruction	Requires meticulous dissection to avoid injury to the deeper quadriceps layers
Allows combined ligament reconstructions (e.g., ACL + ALL/LET or ACL + AOL) using a single graft harvest	Requires familiarity with the harvesting technique and use of a closed tendon stripper
Preserves the hamstring tendons and knee flexor function	Limited number of comparative clinical studies currently available
Does not create a full‐thickness quadriceps tendon defect or require patellar bone harvest	Very long‐term clinical outcomes are still unknown
Preserves future autograft options for revision or additional ligament reconstruction by avoiding harvest of the hamstring tendons and full‐thickness quadriceps tendon	Technique may not be suitable in patients with previous quadriceps tendon injury or surgery

ACL, anterior cruciate ligament; ALL, anterolateral ligament; AOL, anterior oblique ligament; LET, lateral extra‐articular tenodesis.

Over the last decade, quadriceps‐based grafts have progressively gained popularity because of their favorable biomechanical profile and lower donor‐site morbidity compared with traditional autografts. Rather than representing an entirely new graft source, the RF tendon should be considered an evolution of quadriceps tendon harvesting, selectively exploiting the superficial layer while preserving the intermediate and deep components of the extensor mechanism.^7^, ^12^ This selective harvest preserves the remaining quadriceps tendon, avoids violation of the suprapatellar capsule, and maintains future autograft options by leaving both the hamstring tendons and the full‐thickness quadriceps tendon available for possible revision or additional ligament reconstruction procedures.^7^, ^11^, ^12^

Biomechanical evidence further supports the use of the RF tendon. Mestriner et al.^5^ showed that the double‐stranded RF graft exhibits ultimate strength comparable to that of the patellar tendon graft, whereas the single‐stranded configuration shows biomechanical behavior similar to iliotibial band grafts used for anterolateral procedures. These findings support the concept that a single RF harvest may provide sufficient tissue for combined ACL and peripheral ligament reconstruction without requiring additional donor sites. Furthermore, the graft typically measures between 28 and 35 cm, allowing multiple preparation strategies with adequate diameter for primary, revision, and multiligament knee reconstruction.^5^, ^7^

A critical technical aspect of the procedure is identification of the correct dissection plane between the RF tendon and the vastus intermedius approximately 3 cm proximal to the superior pole of the patella. At this level, the tendon layers are more easily distinguished, facilitating maintenance of a superficial dissection plane while reducing the risk of inadvertent arthrotomy that becomes more likely closer to the patellar insertion, where the quadriceps layers progressively converge.^11^ Preservation of the intermediate and deep quadriceps layers also maintains the integrity of the extensor mechanism and may reduce arthroscopic fluid extravasation during concomitant intra‐articular procedures.^7^, ^11^

Early clinical evidence has also been encouraging. Barros et al.^3^ showed that, although quadriceps strength deficits are still present at 6 months after ACL reconstruction using an RF autograft, hamstring strength recovers almost completely, suggesting that postoperative muscle recovery follows the expected pattern observed after quadriceps‐based graft harvesting. More recently, Pineda et al.^4^ reported quadriceps strength recovery at 12 months comparable with that observed after quadriceps tendon and hamstring autografts, showing noninferiority of the RF graft with respect to extensor muscle performance. Likewise, Okutan et al.^6^ showed that RF graft harvest did not result in selective RF muscle volume loss on quantitative magnetic resonance imaging and was associated with limb symmetry indexes exceeding 90% for most strength and functional parameters at 1 year, supporting the concept of limited donor‐site morbidity.

Nevertheless, several technical considerations should be acknowledged. Meticulous identification of the correct surgical plane is essential to avoid graft transection, violation of the deeper quadriceps layers or suprapatellar capsule, and inadequate graft dimensions.^7^, ^11^ Furthermore, despite the growing body of evidence supporting RF tendon harvest, the available literature remains relatively limited and is still composed predominantly of technical notes, biomechanical investigations, and early clinical series. Although recent prospective comparative studies have strengthened the evidence supporting this graft, additional high‐quality randomized studies with long‐term follow‐up are necessary to evaluate graft survivorship, return‐to‐sport outcomes, patient‐reported outcome measures, and donor‐site morbidity.^4^, ^6^

Despite these limitations, the RF tendon appears to represent a promising addition to the contemporary ACL reconstruction armamentarium. Its combination of favorable biomechanical properties, substantial graft length, minimally invasive harvest, preservation of the extensor mechanism, and versatility for combined ligament reconstruction procedures may make it an attractive option for selected primary, revision, and multiligament knee reconstructions. As the available clinical evidence continues to expand, the RF tendon has the potential to become an established alternative among contemporary autograft options.^4^, ^6^, ^12^

## DISCLOSURES

The authors (R.D’., A.M., R.M., A.D.P., E.P., L.M.S., N.U.) declare that they have no known competing financial interests or personal relationships that could have appeared to influence the work reported in this article.

## PATIENT CONSENT

The patient provided written informed consent for the surgical procedure and for the acquisition and publication of intraoperative photographs and video recordings for scientific and educational purposes.
